# Receptor-binding domain 2 of *Clostridioides difficile* binary toxin as a promising vaccine component against *C. difficile* infection

**DOI:** 10.64898/2026.08.30.748174

**Published:** 2026-08-31

**Authors:** Shaohui Wang, Joshua Heuler, Yukihiro Nakanishi, Hyeun Bum Kim, Xingmin Sun

## Abstract

Symptoms of *Clostridioides difficile infection* (CDI) are primarily caused by two major protein toxins, toxin A (TcdA) and toxin B (TcdB). In addition, approximately 5-30% of *C. difficile* strains produce a third toxin, *C. difficile* binary toxin (CDT), which is has been associated with enhanced virulence and severe disease. CDT consists of an enzymatic component CDTa, and a binding and translocation component CDTb, which mediates the delivery of CDTa into host cells. CDTb contains two receptor-binding domains, RBD1 and RBD2. Recent structural studies suggest that RBD2 plays a critical role in the formation and stabilization of the di-heptameric CDTb assembly required for efficient intoxication of host cells. In this study, we evaluated the immunogenicity and protective potential of RBD1 and RBD2 using *in silico, in vitro* and *in vivo* approaches. Sequence analysis demonstrated that RBD2 is highly conserved among diverse CDT-producing *C. difficile* ribotypes and toxinotypes. Immunization of mice with RBD2, but not RBD1 conferred effective protection against direct CDT challenge. Moreover, RBD2 immunization protected hamsters against infection with a CDT-only-producing *C. difficile* strain (DSM 101085; TcdA⁻TcdB⁻CDT⁺). Mechanistically, anti-RBD2 serum, but not anti-RBD1 serum, effectively neutralized CDT-mediated cytotoxicity, as demonstrated by inhibition of cell rounding in Vero cells. Collectively, these findings identify RBD2 as a promising vaccine antigen targeting CDT and provide functional evidence supporting its critical role in CDT-mediated host-cell intoxication. Incorporation of RBD2 into multivalent *C. difficile* vaccines may broaden protection against hypervirulent, CDT-producing strains.

## Full Text Availability

The license terms selected by the author(s) for this preprint version do not permit archiving in PMC. The full text is available from the preprint server.

